# Type C Pancreaticobiliary Maljunction Is Associated With Perforated Choledochal Cyst in Children

**DOI:** 10.3389/fped.2020.00168

**Published:** 2020-04-17

**Authors:** Linlin Zhu, Jing Xiong, Zhibao Lv, Jiangbin Liu, Xiong Huang, Weijue Xu

**Affiliations:** Department of General Surgery, Shanghai Children's Hospital, Shanghai Jiao Tong University, Shanghai, China

**Keywords:** choledochal cyst, perforation, congenital biliary dilatation (CBD), pancreaticobiliary maljunction (PBM), pediatric

## Abstract

**Background:** Perforation of a choledochal cyst (CC) is not rare, but the pathogenesis of spontaneous perforation has not been established. Pancreaticobiliary maljunction (PBM) is commonly seen in association with choledochal cyst. To explore the relationship between PBM and perforated CC, a retrospective study was conducted.

**Methods:** We analyzed all the patients with CC who underwent surgery in our hospital from 2014.06.01 to 2018.12.31. All patients were divided into two groups: group 1 were patients with perforated CC, and group 2 were patients with non-perforated CC. We recalled all the patients records to identify types of PBM. PBM was divided into four types [(A) stenotic type, (B) non-stenotic type, (C) dilated channel type, and (D) complex type] according to the classification proposed by the Committee on Diagnostic Criteria of the Japanese Study Group on Pancreaticobiliary Maljunction (JSGPM) in 2015.

**Results:** There were 186 patients with CC in all, and 116 patients showed PBM. Twenty patients in group 1 and 96 patients in group 2. There was an extremely higher percentage of type C PBM in group 1 than in group 2 (60 and 17.7%, respectively). More fusiform dilatation cases were found in group 1 (70%) than in group 2 (58.3%). Also there were more type C PBM in fusiform cases and type A PBM were frequently seen in cystic cases (*P* < 0.01).

**Conclusions:** We found that Type C PBM and fusiform common bile duct maybe relate to the perforation of choledochal cyst. Patients with type C PBM and fusiform common bile duct should be treated more proactively, preferably before they perforate.

## Introduction

Choledochal cyst (CC) is a rare congenital disorder characterized by dilatation of the bile duct. It occurs in one out of 1,000 persons in Asia, while less frequently in one out of 50,000–150,000 individuals in the West, with the predominance of females over males being 3:1 (1, 2).

CC is mostly associated with pancreaticobiliary maljunction (PBM), where the biliary and pancreatic ducts converge outside the duodenal wall beyond the confluence of the sphincter of Oddi. This abnormal anatomical communication allows 2-way regurgitation of pancreaticobiliary and biliopancreatic reflux and has serious clinical sequelae such as pancreatitis and cholangitis, and possibly the development of biliary carcinoma over the long term (3). Various classifications of PBM according to the form of the confluence between the distal common bile duct and the pancreatic duct have been created (1, 4, 5). In 2015 the Committee on Diagnostic Criteria of the Japanese Study Group on Pancreaticobiliary Maljunction (JSGPM) proposed a new PBM classification, and in 2017 Urushihara et al. showed that this new classification was simple and correlated well with clinical features (6).

Perforation of a choledochal cyst (CC) is not rare, with a reported frequency ranging from 1.8 to 18% (7–10). The pathogenesis of spontaneous perforation has not been established, but proposed mechanisms include reflux of pancreatic secretions (8), increased intraluminal pressure due to protein plugs (7, 11), and viral infection (12). PBM is the cause of regurgitation between the bile and pancreatic ducts. Different types of PBM may lead to varying degrees of reflux of pancreatic secretions and intraluminal pressure.

In this report, we aim to explore the relationship between types of PBM and perforated CC. This would provide us with a better understanding on the possible factors that contribute to perforation that will facilitate a more timely diagnosis and management.

## Materials and Methods

**Figure 1:**
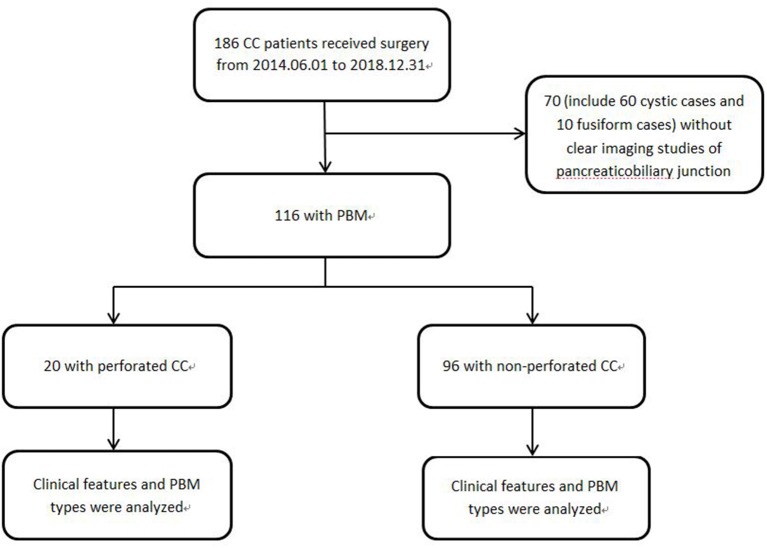
Study profile 70 patients who could not be found clear PBM images either in MRCP nor in IOCP were excluded.

This study was approved by the Institutional Review Board of our Hospital (NO. 2018RY028). We retrospectively reviewed all the medical records of patients with CC who underwent surgery in our hospital from 2014.06.01 to 2018.12.31, including medical histories, operation notes and imaging data. Patients without clear imaging studies of the common bile duct, pancreatic duct, and pancreaticobiliary junction were excluded from this study. All patients were divided into two groups: group 1 were patients with perforated CC, and group 2 were patients with non-perforated CC (Figure 1). Using the following imaging methods: magnetic resonance cholangiopancreatography (MRCP) and intraoperative cholangiopancreatography (IOCP), common bile duct types were defined as cystic type (Todani Ia and Ib, Todani VIa with length-width ratio <2) and fusiform type (Todani Ic, Todani VIa with length-width ratio more than 2); and PBM was divided into four types [(A) stenotic type, (B) non-stenotic type, (C) dilated channel type, and (D) complex type] according to the classification proposed by the Committee on Diagnostic Criteria of the Japanese Study Group on Pancreaticobiliary Maljunction (JSGPM) in 2015.

## 2015 Classification of PBM (Figure 2)

Type A (stenotic type): The stenotic segment of the distal common bile duct joins the common channel, and dilatation of the common bile duct is seen.

**Figure 2:**
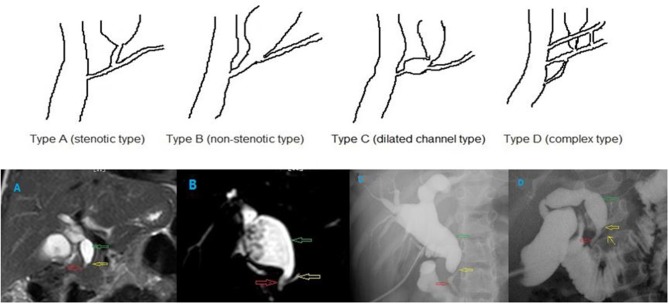
Different types of PBM. **(A)** stenotic type, **(B)** non-stenotic type, **(C)** dilated channel type, and **(D)** complex type. The common channel (the red hollow arrow), the pancreatic duct (the yellow hollow arrow), and the dilated bile duct (the green hollow arrow). In the type D PBM, there was a accessory pancreatic duct (the yellow arrow).

Type B (non-stenotic type): The distal common bile duct without any stenotic segment smoothly joins the common channel. Localized dilatation of the common channel is not seen.

Type C (dilated channel type): The common channel is dilated. The narrow segment of the distal common bile duct joins the common channel, and abrupt dilatation of the common channel is seen.

Type D (complex type): Complicated union of the pancreaticobiliary ductal system as follows: PBM associated with annular pancreas, pancreas divisum, or other complicated duct systems.

## Statistical Analysis

The collected data were organized, tabulated, and statistically analyzed using Statistical Package for Social Science (SPSS) version 16 (SPSS Inc., USA). Qualitative data, frequency, and percent distribution were calculated, and Chi square test was used for comparison between groups. Quantitative data, mean, and range were calculated, and for comparison between two groups, the independent samples (*t*) test and ANOVA were used. For interpretation of results, *P* < 0.05 was considered significant.

## Result

**Figure 3:**
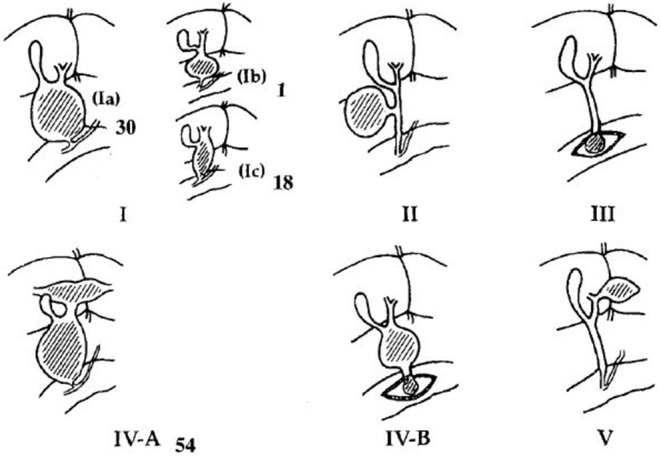
Todani's classification of choledochal cyst.

A total of 186 patients with choledochal cyst who underwent surgery in our hospital from 2014.06.01 to 2018.08.31 were reviewed, and 70 patients without clear common channel images were excluded (Figure 1). All the choledochal cysts were type I and type VIa according to Todani's classification (Figure 3) (14). There were no typeII and typeIII CC. There were 20 patients with perforated choledochal cyst (group 1), and 96 patients with non-perforated choledochal cyst (group 2). These patients included 27 males and 89 females, and the median age at operation was 3.7 years (IQR: 1 month−13 years).

Baseline demographic features were similar between the two groups (Table 1). Female predominance was found in both groups (75 and 77.1%) The median age at operation in group 1 and in group 2 was 3.3 and 3.8 years, respectively. More than 1/3 patients were between 2 and 3 years old, both in group 1 (7/20, 35.0%) and in group 2 (33/96, 34.2%). Within all 186 patients, group 2 contains more infants than group 1 (21.11 vs. 12%). Although there was no statistically significant difference, infants did not appear to be susceptible to choledochal cyst perforation (*P* = 0.18).

**Table 1:** Demographics of children with pancreaticobiliary maljunction.

	**Group 1**	**Group 2**	**Total**	***P*-value**
Gender				0.84
Male	5 (25%)	22 (22.9%)	27 (23.3%)	
Female	15 (75%)	74 (77.1%)	89 (76.7%)	
Age at operation (range)	3.3 y (9 m−7 y)	3.8 y (1 m−13 y)	3.7 y (1 m−13 y)	0.45
Clinical features				<0.01
Prenatal diagnosis	0	17 (17.7%)	17 (14.7%)	0.09
Abdominal pain	13 (65%)	51 (53.1%)	64 (55.2%)	0.33
Vomiting	16 (80%)	35 (36.5%)	51 (44.0%)	0.00
Abdominal mass	0	3 (3.1%)	3 (2.6%)	1.00
Jaundice	2 (10%)	28 (29.2%)	30 (25.9%)	0.07
Laboratory findings				
Serum amylase, U/L (IQR)	178.8 (26–1,219)	278.3 (2–1,830)	263.1 (2–1,830)	0.40
Bile amylase, U/L (IQR)	7,548.7 (10–323,400)	53,351.1 (1–601,600)	44,988.7 (1–601,600)	0.98
Type of common bile duct^*^				0.33
Cystic	6 (30%)	40 (41.7%)	46 (39.7%)	
Fusiform	14 (70%)	56 (58.3%)	70 (60.3%)	
Bile duct stones	9 (45%)	40 (41.7%)	49 (42.2%)	0.79

* *In all patients with CC, there were nine cystic cases and 16 fusiform cases in Group 1; in Group 2, there were 97 cystic cases and 64 fusiform cases, P = 0.02. There were significant differences between the two groups*.

Clinical symptoms were different between the two groups (*P* < 0.01). There were more patients presented with vomiting in group 1 than in group 2 (80 vs. 36.5%). Though without significant difference, prenatal diagnosis and jaundice were more frequently seen in group 2 (*P* = 0.09, *P* = 0.07, respectively).

More fusiform dilations were found in group 1 than in group 2 (70.0 vs. 58.3%, *P* = 0.33). As Figure 1 showed, 60 cystic cases (57 in group 1) were excluded, while only 10 fusiform cases were without clear PBM images. The small common bile ducts' diameters allowed a high PBM visualization rate. Within all patients with CC, either with or without clear PBM images, there were much more cystic cases than fusiform cases in group 2. So the percentage of fusiform cases were significantly higher in group 1 than in group 2, *P* = 0.02. There was no significant difference between the two groups in bile duct stones/protein plugs (Table 1).

There were more type C PBMs in group 1, and more type A PBMs in group 2, both with significant differences (*P* < 0.01). No differences in type B and D PBMs between the two groups (*P* = 0.18, *P* = 0.28, respectively) (Table 2, Figure 2).

**Table 2:** Types of PBM in Group 1 and Group 2.

**Types of PBM**	**Group 1 (%)**	**Group 2 (%)**	***P***
A	3 (15)	48 (50)	<0.01
B	2 (10)	26 (27.1)	0.18
C	12 (60)	17 (17.7)	0.00
D	3 (15)	5 (5.2)	0.28
Total	20	96	<0.01

*There were more type C PBM in group 1, and more type A PBM in group 2*.

We analyzed PBM types according to different common bile duct type. There were different PBM types in fusiform and cystic cases. Fusiform cases comprised more type B and C PBMs (*P* = 0.02, *P* < 0.01, respectively), while type A PBMs were predominant in cystic cases (*P* = 0.00). There was no big difference of type D PBMs between fusiform and cystic cases (*P* = 0.21) (Table 3).

**Table 3:** Types of PBM in fusiform and cystic cases.

	**Fusiform (%)**	**Cystic (%)**	***P***
A	16 (22.9)	35 (76.1)	0.00
B	22 (31.4)	6 (13)	0.02
C	25 (35.7)	4 (8.7)	<0.01
D	7 (10)	1 (2.2)	0.21
Total	70 (100)	46 (100)	<0.01

*Fusiform cases contained more type C and B PBMs, while there were more type A PBMs in cystic cases*.

There were no significant differences in serum amylase and bile amylase between patients in group 1 and patients in group 2 (*P* = 0.40, *P* = 0.98, respectively, Table 1). When analyzed according to different PBM types, there were no significant differences in patients' serum amylase and bile amylase between the two groups (*P* > 0.05).

## Discussion

To our knowledge, this is the first report focused on the relationship between PBM types and perforated choledochal cysts, and this report represented the largest study to date on perforated CC.

Choledochal cysts were first described by Vater and Ezler in 1723 (13). With the standard surgery of complete extrahepatic bile duct cyst excision and Roux-en-Y hepaticojejunostomy bilioenteric reconstruction, the outcomes of CC patients are very good. However, spontaneous biliary perforation could be fatal and it is not rare. In our series, the perforation rate was 11.9%, which is consistent with literature reports.

CC perforation may manifest clinically as an acute abdomen with bile peritonitis. Previous authors had described abdominal pain and vomiting as the most common presentation (8, 10). This is also observed in our series with vomiting as the ubiquitous complaint. However, abdominal pain was the most common symptom in non-perforated cases. Jaundice and abdominal mass were both common in non-perforated cases, probably because they are frequently observed in cystic cases.

There was no gender difference between group 1 and group 2. Female patients presented primarily in both groups. The median age at operation was similar in group 1 and group 2. The predilection age in both groups was 2~3 years old, which was consistent with Ngoc et al. (15). However, within all the 186 patients, there were more infants in group 2, probably due to the cystic forms is more frequently seen in infants. Perforation is prone to occurred in older patients, which was consistent with Chiang et al. (10). The reason of this tendency is probably that older patients are frequent with fusiform common bile ducts.

We found close relationship between type C PBM and perforated CC, as supported by the finding that there were 60% of type C PBM in group 1, while only 17.7% type C PBM were found in group 2. Urushihara et al. (6) also found a similar result. They reported a high incidence of biliary perforation in patients with type C PBM. They found that the incidence of protein plugs was high in patients with type C. And they thought that the cause of biliary perforation is probably biliary obstruction due to protein plugs, in which pancreatic juice easily regurgitates into the bile duct, resulting in severe inflammation in the wall of the bile duct. But they did not define the protein plugs. In our study, we found no difference in the incidence of bile duct stones/protein plugs (defined as stones in the common channels) between the two groups. Furthermore, no significant differences were found in the level of bile amylase between the two groups and among different types of PBM. These results failed to indicate any relationship between pancreatic juice regurgitation and CC perforation. The small sample of perforated cases limited the result, thus we need more cases to verify it.

Our study also provided other major findings. There were more fusiform common bile ducts in group 1 than in group 2. In this study, we excluded the patients without clear common channel images. Clear common channel images relate closely to the diameter of cysts. The larger the cyst was, the harder to get a clear common channel image. In fusiform cases the average diameter of cysts was 1.58 cm, while in cystic cases was 4.36 cm. Thus, more cystic cases in group 2 were excluded from the study. When all cases were included, there was higher percentage of cystic cases in group 2 than in group 1 with significant difference. This result was consistent with Ando et al. (7) and Fukuzawa et al. (11) reports. The mechanism of high perforation rate in fusiform cases is still unknown. In this study, we found that the types of PBM were different in fusiform and cystic choledochal cysts (*P* < 0.01). Type A PBM were frequently seen in cystic cases while in fusiform cases more type C PBM were found. PBM is accepted as one of the causes of choledochal cyst, as it creates a nidus for reflux of pancreatic enzymes into the common bile duct that causes damage to the ductal wall and leads to cyst formation (16, 17). The results of this study indicate that maybe different types of PBM result in different types of abnormal common bile ducts. Therefore, the higher perforation rate in patients with fusiform choledochal cyst may be caused by the higher proportion of C-type PBM in fusiform cases. However, Ando et al. (8) described 13 cases of perforated CBD and reported that the shape of the common bile duct was cystic in 8 and fusiform in 5 cases. This didn't agree with our finding. More data should be collected and evaluated regarding the shape of the common bile duct as a risk factor.

Timing of surgery for prenatally-diagnosed CCs is controversial, some have advocated for repair within the first several months of life while others suggest a delayed surgical procedure in asymptomatic cases (18–21). When encountering a patient with fusiform common bile duct or type C PBM confirmed by ultrasound or MRCP, whether prenatally-diagnosed or not, a proactive surgical approach should be considered, preferably before they perforate (13, 15).

The main limitation of this study is the low visualization rate of PBM. The visualization rate of PBM relates to the diameter of cyst. As we know, more cystic cases in group 2 were excluded and type A PBM was primary in cystic cases, we can speculate that more cases with type A PBM in group 2 were excluded. If we include all the patients, the differences of type C PBM and fusiform rate between the two groups will be even more obvious. The low visualization rate of PBM won't affect the results.

## Conclusions

The incidence of type C PBM and fusiform common bile ducts were higher in perforated choledochal cyst than in non-perforated choldohcal cyst, indicating that there may be a relationship between type C PBM/fusiform common bile ducts and choledochal cyst perforation. Patients with type C PBM and fusiform common bile duct should be treated more proactively to prevent perforation.

## Data Availability Statement

All datasets generated for this study are included in the article/supplementary material.

## Ethics Statement

The studies involving human participants were reviewed and approved by Institutional Review Board of Shanghai Children's Hospital. Written informed consent from the participants' legal guardian/next of kin was not required to participate in this study in accordance with the national legislation and the institutional requirements.

## Author Contributions

LZ and WX contributed to study conception and design. JX and XH contributed to data acquisition. ZL and LZ contributed to analysis and data interpretation. LZ and JL contributed to drafting of the manuscript, while WX contributed to critical revision.

## Conflict of Interest

The authors declare that the research was conducted in the absence of any commercial or financial relationships that could be construed as a potential conflict of interest.
